# Influence of apico‐coronal positioning of tissue‐level implants on marginal bone stability during supracrestal tissue height establishment: A multi‐center prospective study

**DOI:** 10.1111/cid.13128

**Published:** 2022-08-24

**Authors:** Sergio Spinato, Fabio Bernardello, Teresa Lombardi, Carlo Maria Soardi, Marcello Messina, Davide Zaffe, Claudio Stacchi

**Affiliations:** ^1^ Private Practice Sassuolo Modena Italy; ^2^ Private Practice Terranegra di Legnago Verona Italy; ^3^ Department of Health Sciences Magna Græcia University Catanzaro Italy; ^4^ Private Practice Brescia Italy; ^5^ Private Practice Trieste Italy; ^6^ Department of Biomedical, Metabolic and Neural Sciences University of Modena and Reggio Emilia Modena Italy; ^7^ Department of Medical, Surgical and Health Sciences University of Trieste Trieste Italy

**Keywords:** bone remodeling, marginal bone loss, soft tissue thickness, supracrestal tissue height, tissue‐level implants

## Abstract

**Introduction:**

Supracrestal tissue height establishment is a crucial factor influencing peri‐implant marginal bone modifications prior to prosthesis delivery. If mucosal thickness is insufficient, peri‐implant marginal bone resorption occurs to allow appropriate supracrestal tissue height formation. This study evaluates if marginal bone resorption occurring around tissue‐level implants before prosthetic loading could be compensated by adapting apico‐coronal positioning to mucosal thickness.

**Methods:**

Patients requiring placement of one single implant in the posterior mandible were treated with tissue‐level implants with a 3‐mm high transmucosal machined component and moderately rough implant body. Based upon vertical mucosal thickness measured after buccal flap reflection, implants were placed with the treated part: (group 1) 2 mm below crestal level in presence of thin mucosa (<2.5 mm); (group 2) 1 mm below the crestal level in presence of medium mucosa (2.5–3.5 mm); (group 3) at equicrestal level in presence of thick mucosa (>3.5 mm).

**Results:**

Forty‐nine implants, placed in 49 patients were included in final analysis (group 1: 18 implants; group 2: 16 implants; group 3: 15 implants). Mean marginal bone resorption after 5 months of healing was 0.66 ± 0.49 mm, 0.32 ± 0.41 mm, and 0.22 ± 0.52 mm in groups 1, 2, and 3, respectively. Inter‐group analysis highlighted significant differences between the three groups after ANOVA test (*p* = 0.025). However, adaptation of apico‐coronal implant positioning in relation to mucosal thickness, allowed to avoid early exposure of the treated surface in 100%, 93.7%, and 53.3% of the implants in groups 1, 2, and 3, respectively.

**Conclusion:**

During supracrestal tissue height formation, tissue‐level implants inserted adapting apico‐coronal positioning in relation to mucosal thickness exhibited greater marginal bone resorption at sites with thin mucosa than at sites with medium or thick mucosa. However, anticipating supracrestal tissue height establishment by adapting apico‐coronal implant positioning in relation to mucosal thickness may effectively prevent unwanted exposure of treated implant surface.

**What is known**

- Early marginal bone loss >0.5 mm during the first year of function represents a risk factor for future peri‐implantitis development.
- Supracrestal tissue height establishment is a crucial factor influencing peri‐implant marginal bone modifications prior to prosthetic loading.
- If mucosal thickness is insufficient, peri‐implant marginal bone resorption occurs to allow appropriate supracrestal tissue height formation.

**What this study adds**

- Anticipating supracrestal tissue height establishment by adapting apico‐coronal positioning of tissue‐level implants in relation to mucosal thickness may effectively prevent early unwanted exposure of treated implant surface.

## 1 INTRODUCTION

Stability of marginal bone levels has always been considered fundamental to evaluate long‐term dental implant efficacy. Radiographic marginal bone loss up to 1.5–2 mm during the first year of function and a maximum of 0.2 mm annually thereafter, is a traditionally accepted criterion to define implant success.^1^, ^2^, ^3^, ^4^, ^5^, ^6^

Early marginal bone loss (EMBL) is a non‐infective peri‐implant crestal bone remodeling process occurring within the first year of function.^7^ EMBL has a multi‐factorial etiology, being influenced by various surgical and prosthetic factors including insufficient crestal width,^8^, ^9^, ^10^, ^11^ surgical trauma,^12^ supracrestal tissue height formation,^13^, ^14^ microbial colonization of implant‐abutment micro‐gap,^15^, ^16^ presence of horizontal implant‐abutment mismatch (“platform‐switching”),^17^, ^18^, ^19^ number of abutment connections/disconnections,^20^ prosthetic abutment height,^21^, ^22^, ^23^, ^24^, ^25^, ^26^ implant‐abutment connection design and mechanical stability and adaptive response to occlusal loading.^7^

Recent studies highlighted the importance of limiting EMBL to improve dental implant clinical outcomes. Galindo‐Moreno and colleagues observed that EMBL >0.44 mm after 6 months of prosthetic loading is a strong predictor of >2 mm of marginal bone loss at 18‐month follow‐up.^27^ In a recent 10‐year prospective study, Windael and colleagues demonstrated that implants with EMBL ≥0.5 mm showed 5.43 times higher odds of future peri‐implantitis development than implants with EMBL <0.5 mm during the first year of function.^28^

Supracrestal tissue height formation is a principal factor influencing peri‐implant marginal bone adaptation processes prior to prosthesis delivery. When the implant becomes exposed to the oral cavity, soft tissues establish a “cuff‐like” barrier sealing the trans‐epithelial component of the fixture. Pre‐clinical studies by Abrahamsson and colleagues^29^ and Berglundh and Lindhe^30^ suggested that a minimum mucosal thickness is required to establish correct epithelial‐connective tissue attachment. If mucosal thickness is insufficient, peri‐implant marginal bone resorption occurs to allow appropriate supracrestal tissue height formation. Further clinical studies by Linkevicius and colleagues confirmed these findings in humans, suggesting that mucosal thickness is a significant influencing factor on peri‐implant marginal bone stability.^31^, ^32^

Subcrestal implant positioning was originally proposed as a clinical strategy to compensate possible reduction of peri‐implant marginal bone levels during the first year of function.^33^ However, studies performed in dogs using two‐piece implants showed that the more subcrestal the micro‐gap position, the greater the marginal bone loss.^13^, ^34^ Even if deeper implant insertion does not limit marginal bone resorption,^13^, ^35^ adaptation of apico‐coronal implant placement may prevent colonization of the treated implant surface by the oral bacterial biofilm. In this regard, Linkevicius and colleagues distinguished marginal bone resorption occurring around subcrestal implants into two different components: bone remodeling (bone resorption occurring above the implant neck) and bone loss (bone resorption exposing implant neck and/or the underlying implant surface).^36^

Unlike two‐piece implants which present a microgap at crestal bone level, tissue‐level implants have no gap in this region.^37^ The absence of this gap seems to influence supracrestal tissue height formation around tissue‐level implants.^38^ However, only limited evidence exists of the relationship between apico‐coronal positioning of tissue‐level implants, EMBL and vertical mucosal thickness.

The present multi‐center prospective study aims to evaluate if EMBL occurring around tissue‐level dental implants before prosthesis delivery could be compensated by adapting apico‐coronal positioning to mucosal thickness.

## 2 MATERIALS AND METHODS

### 2.1 Experimental design

This multi‐center observational prospective study was reported following STROBE (Strengthening the Reporting of Observational studies in Epidemiology) guidelines. All procedures were in full accordance with the principles outlined in WMA Helsinki Declaration and following modifications (Fortaleza 2013).^39^ The study protocol was approved by the relevant Ethical Committee (Regione Calabria, Sezione Area Centro, Nr. 370/2020), and was recorded in a public register of clinical trials (www.clinicaltrials.gov—NCT05363306). After thorough discussion, all selected patients signed an informed consent in which all clinical procedures, possible risks and therapeutic alternatives were detailed. Patients consented to use of their personal data for research purposes.

All the clinical centers participated to a calibration meeting prior to the study to discuss study protocol and standardize collection of experimental parameters in order to obtain acceptable inter‐examiner consistency.

### 2.2 Patient selection

All patients, selected consecutively from a pool, were treated in six clinical centers. Patients were partially edentulous and required placement of at least one single implant in pristine bone in the posterior mandible. In case of multiple implants, only the most mesial implant was evaluated such that each patient contributed to the study with only one implant.

General inclusion criteria were: (i) age > 18 years; (ii) good general health; (iii) absence of systemic diseases affecting bone metabolism and wound healing; (iv) no regular medication consumption for at least 3 months prior to treatment; (v) patient willingness and capability to fully comply with the study protocol; (vi) written informed consent given.

Local inclusion criteria are: (i) presence of keratinized mucosa at implant site with a minimum bucco‐lingual width of 3 mm; (ii) bone crest at implant site with a minimum of 6 mm width and 9 mm height above the mandibular canal, with no previous or concomitant bone augmentation procedure; (iii) healed bone crest (at least 6 months elapsed from tooth extraction/loss); (iv) presence of opposing dentition.

Exclusion criteria are: (i) history of head or neck radiation therapy; (ii) uncontrolled diabetes (HBA1c >7.5%); (iii) active infections; (iv) immunocompromised patients (HIV infection or chemotherapy within the past 5 years); (v) present or past treatment with intravenous bisphosphonates; (vi) patient pregnancy or lactating at any time during the study; (vii) poor oral hygiene and motivation (full mouth plaque score [FMPS] >25%); (viii) untreated periodontal disease; (ix) psychological or psychiatric problems; (x) alcohol or drug abuse; (xi) participation in other studies, if the present protocol could not be properly followed; (xii) peak insertion torque >60 Ncm.

Before implant placement, patients received oral hygiene instruction and professional deplaquing 1 week before surgery.

### 2.3 Surgical procedures

After administration of 4% articaine solution with adrenaline 1:100 000, a mid‐crestal incision along the center of the edentulous bone ridge was performed. A full‐thickness flap was elevated in two phases as described elsewhere.^25^

1. After full thickness elevation of the buccal flap, a soft‐tissue probe (SSL, Medesy) was used at the center of the future implant site to measure the vertical height of the unseparated lingual flap;
2. full thickness elevation of the lingual flap was then performed exposing the alveolar crest.

**FIGURE 1:**
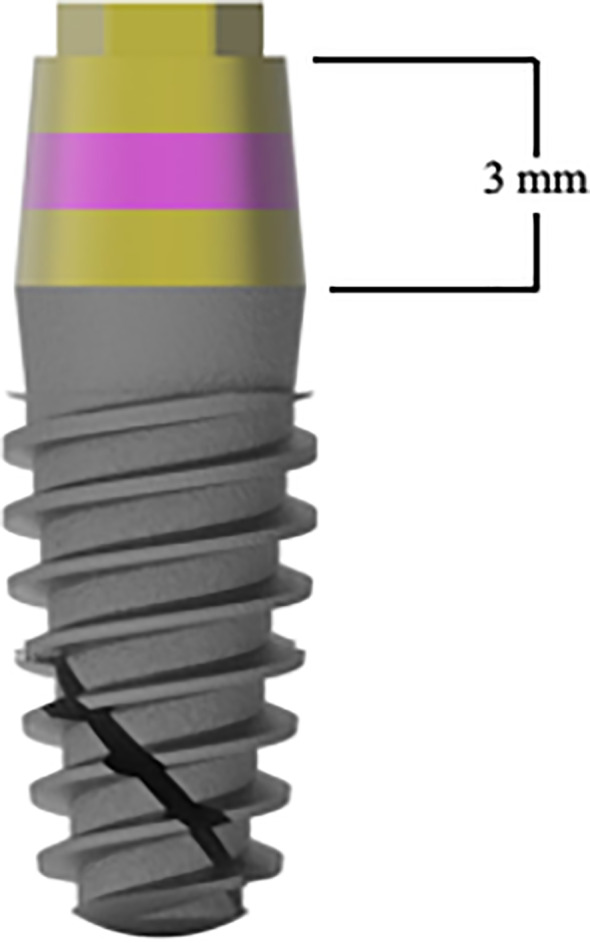
The tissue‐level implant used in the present study. The machined 3‐mm high transmucosal portion presents different colors to sink implants to different apico‐coronal depths

A one‐stage protocol was adopted adhering to manufacturer's recommendations. Sites were prepared for insertion of a tissue‐level implant with a 3‐mm high convergent machined transmucosal component, moderately rough implant body and external hex connection (i‐Smart, i‐Res) at three different crestal levels (Figure 1).

**FIGURE 2:**
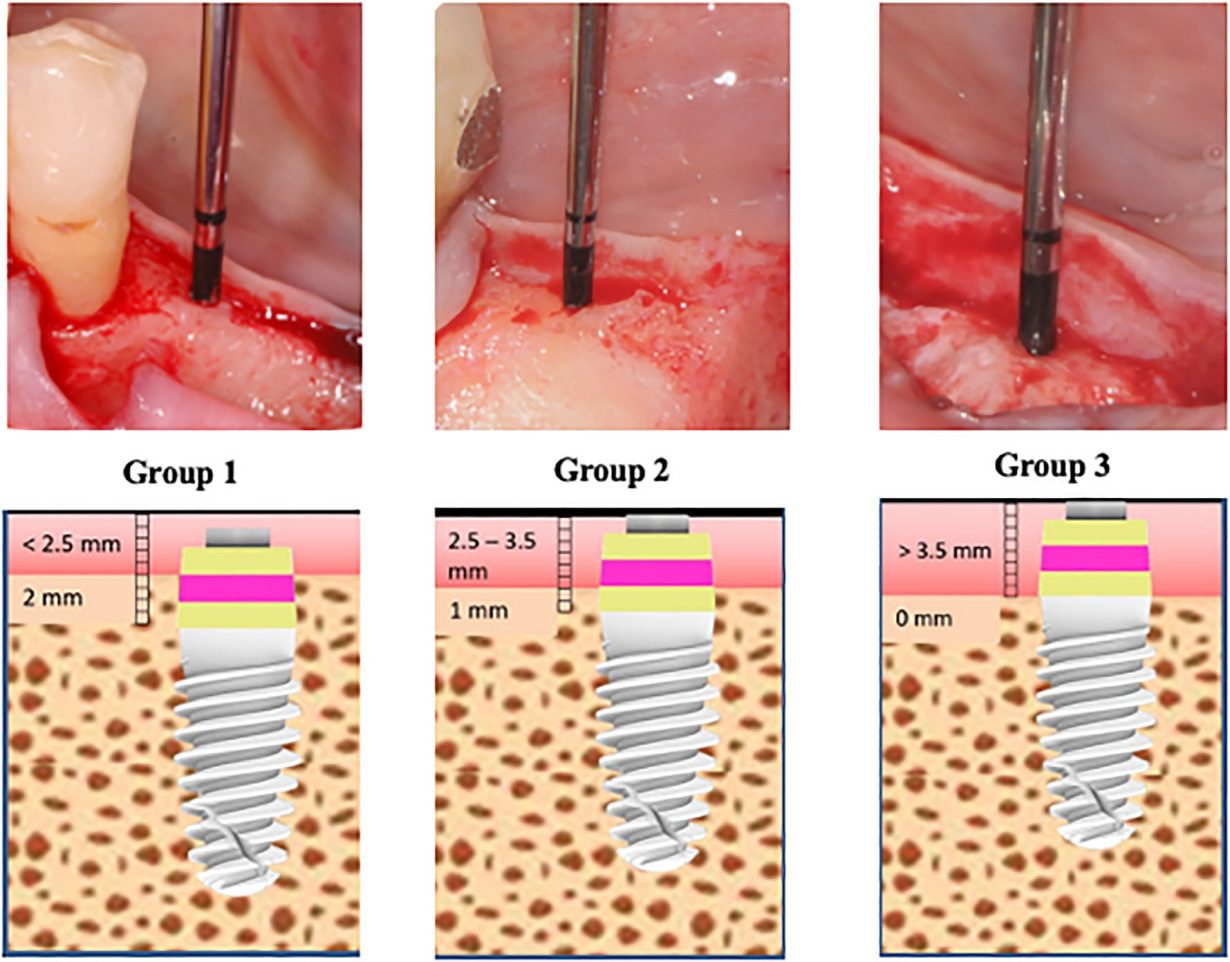
Implants were placed based upon vertical mucosal thickness measured after buccal flap reflection: group 1 (thin mucosa <2.5 mm) with transmucosal portion 2 mm below crestal level; group 2 (medium mucosa between 2.5 and 3.5 mm) with transmucosal portion 1 mm below crestal level; group 3 (thick mucosa >3.5 mm) with transmucosal portion at equicrestal level

Based upon vertical mucosal thickness measured after buccal flap reflection, implants were placed with the treated part: (group 1) 2 mm below crestal level in presence of thin mucosa (<2.5 mm); (group 2) 1 mm below the crestal level in presence of medium mucosa (2.5–3.5 mm); (group 3) at equicrestal level in presence of thick mucosa (>3.5 mm) (Figure 2).

Owing to crest width, all implants were 4.1 mm in diameter. Operators selected appropriate implant lengths (8 or 10 mm) according to available bone height. All implants were left unsubmerged and were covered with a healing cap. Flaps were sutured with synthetic mono‐filament around the transmucosal component. Patients were prescribed antibiotic therapy (amoxicillin 1 g twice a day) for 6 days and non‐steroidal anti‐inflammatory drugs (ibuprofen 600 mg), when needed. Sutures were removed 12–14 days after surgery. No removable prostheses were utilized during the healing period.

After 5 months of healing, patients were referred to prosthodontists for subsequent rehabilitation.

### 2.4 Radiographic measurements

**FIGURE 3:**
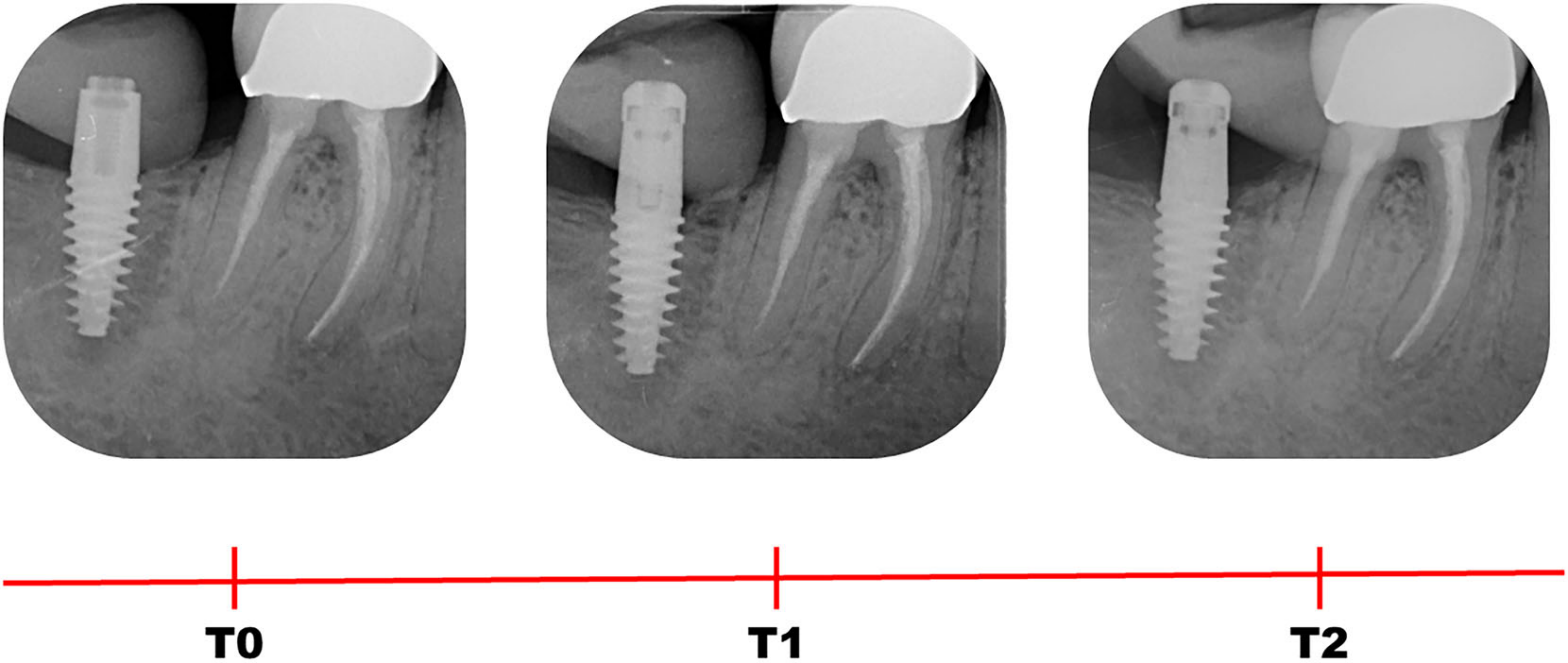
Illustrative case of a group 3 implant (thick mucosa): periapical radiographs, customized with patient‐specific bite jig, were taken at implant placement (T0), 3 months after implant placement (T1), and 5 months after implant placement, immediately before impression taking (T2)

Digital radiographs, customized with patient‐specific bite jigs, were taken using a long‐cone paralleling technique with a Rinn‐type film holder at implant placement (baseline, T0), 3 months after implant placement (T1), and 5 months after implant placement, immediately before impression taking (T2) (Figure 3). All radiographs were performed using the same X‐ray generator technology (FOCUS, KaVo), set to the same parameters (60 kV, 7 mA).

Peri‐implant bone levels (PBLs) were calculated on each radiograph as the linear measurement of the distance between two points: the most coronal point of the implant platform and the most coronal bone‐to‐implant contact. Measurements were corrected referring to the known height and diameter of each implant. The vertical distance between the most coronal point of the implant platform and the most coronal bone‐to‐implant contact was measured on both mesial and distal aspects of the implant at T0 (implant placement), T1 (3 months after implant placement) and T2 (5 months after implant placement).

Therefore, an increased vertical distance between the implant platform reference point and the most coronal bone‐to‐implant contact is considered indicative of bone resorption, whilst a decrease in distance is considered indicative of bone gain.

**FIGURE 4:**
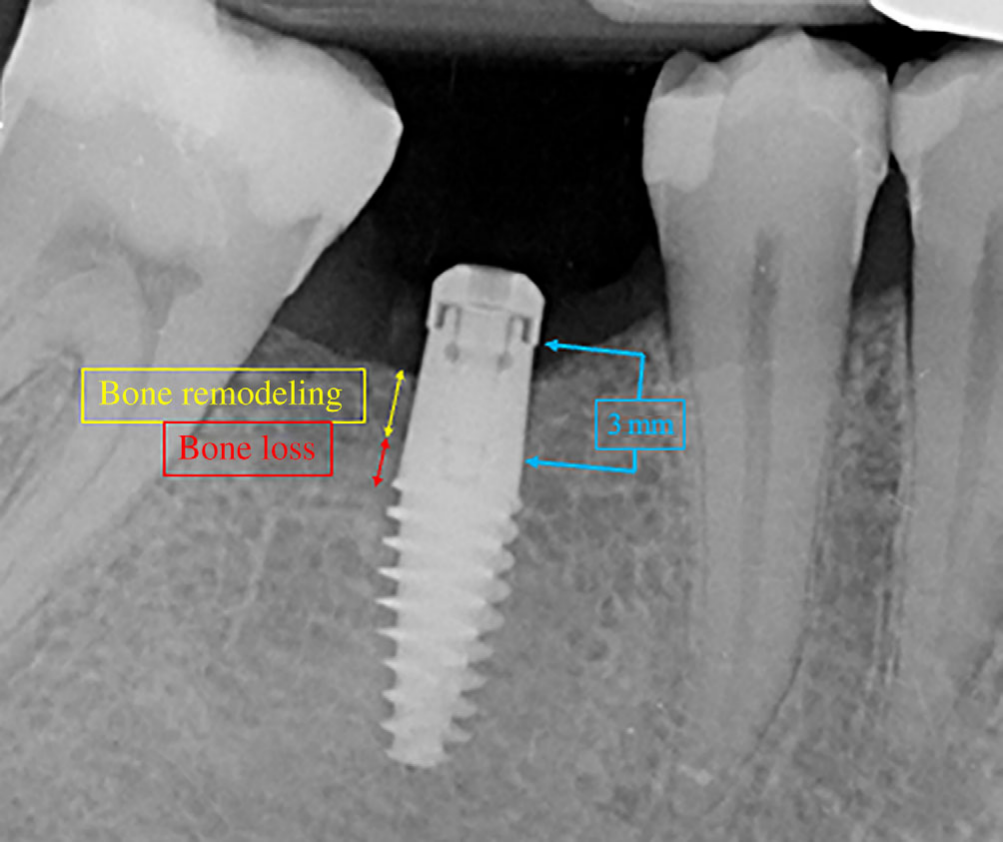
BR: marginal bone resorption occurring around 3‐mm high transmucosal portion of implant, when partially sunk below bone level (groups 1 and 2) (yellow arrow). BL: marginal bone resorption exposing moderately rough implant surface, below the 3‐mm high transmucosal portion (red arrow). BL, bone loss; BR, bone remodeling

In addition, PBL variations were differentiated into two distinct components, as suggested elsewhere (Figure 4).^36^

1. Bone remodeling (BR): marginal bone resorption occurring around the 3 mm high transmucosal portion of the implant, when partially sunk under bone level (groups 1 and 2).
2. Bone loss (BL): marginal bone resorption exposing the treated surface of the implant body, below the 3 mm high transmucosal portion.

Radiographs demonstrating deformation, darkness, and/or other problems were immediately repeated. All measurements were made by two calibrated examiners, blind to mucosal thickness, using a 30‐inch LED‐backlit color diagnostic display with Kodak Digital Imaging Software (Kodak, Eastman Kodak). Each measurement was repeated three times at three different time points as proposed by Gomez‐Roman and Launer.^40^ Examiner calibration was performed by measuring PBL on a sample of 10 radiographs not included in the study. Cohen's *k* coefficient for intra‐examiner and inter‐examiner agreement was 87.2% and 81.6%, respectively, for linear measurements within ±0.1 mm.

### 2.5 Statistical analysis

Statistical analysis was performed by means of *Primer of Biostatistics* (6th Ed.) software.^41^ The patient was considered as the statistical unit (one implant per patient). Considering three treatment group comparisons, a sample of 15 patients from each group was required to detect significant differences (confidence level 5% with statistical power of 80%), with an expected difference in MBL of 0.3 mm (±0.25 mm).^42^ Data for descriptive statistics were expressed as mean ± standard deviation, and were analyzed using the one‐way ANOVA test. Simple linear regression was used to analyze trends. Overall analysis for coincidence was performed to compare regression lines.^24^ The null hypothesis (no difference in MBL among groups) was rejected for a critical significance level of *p* < 0.05.

## 3 RESULTS

A total of 54 consecutively selected patients were enrolled and treated in six clinical centers (SS *n* = 10 patients; FB *n* = 8 patients; TL *n* = 9 patients; CMS *n* = 9 patients; MM *n* = 8 patients; CS *n* = 10 patients). Four group 2 patients did not present at follow‐up visits, being excluded from the study. One group 3 patient was excluded from final analysis due to peak insertion torque exceeding 60 Ncm during implant placement.

The remaining 49 implants, placed in 49 patients (25 males and 24 females, mean age 56.7 ± 11.8 years, range 34–85 years), were included in final analysis (group 1: 18 implants; group 2: 16 implants; group 3: 15 implants). No significant difference between groups based upon age, gender, smoking habits, or history of periodontitis was demonstrated (*p* > 0.05). All implants were successfully osseointegrated at T2 and no complications or adverse events were recorded during the healing period.

### 3.1 Radiographic measurements

No significant difference was demonstrated between mesial and distal PBL within the three groups at any time point. Hence, a single PBL value (the mean of mesial and distal measurements) was calculated for each individual implant.

**FIGURE 5:**
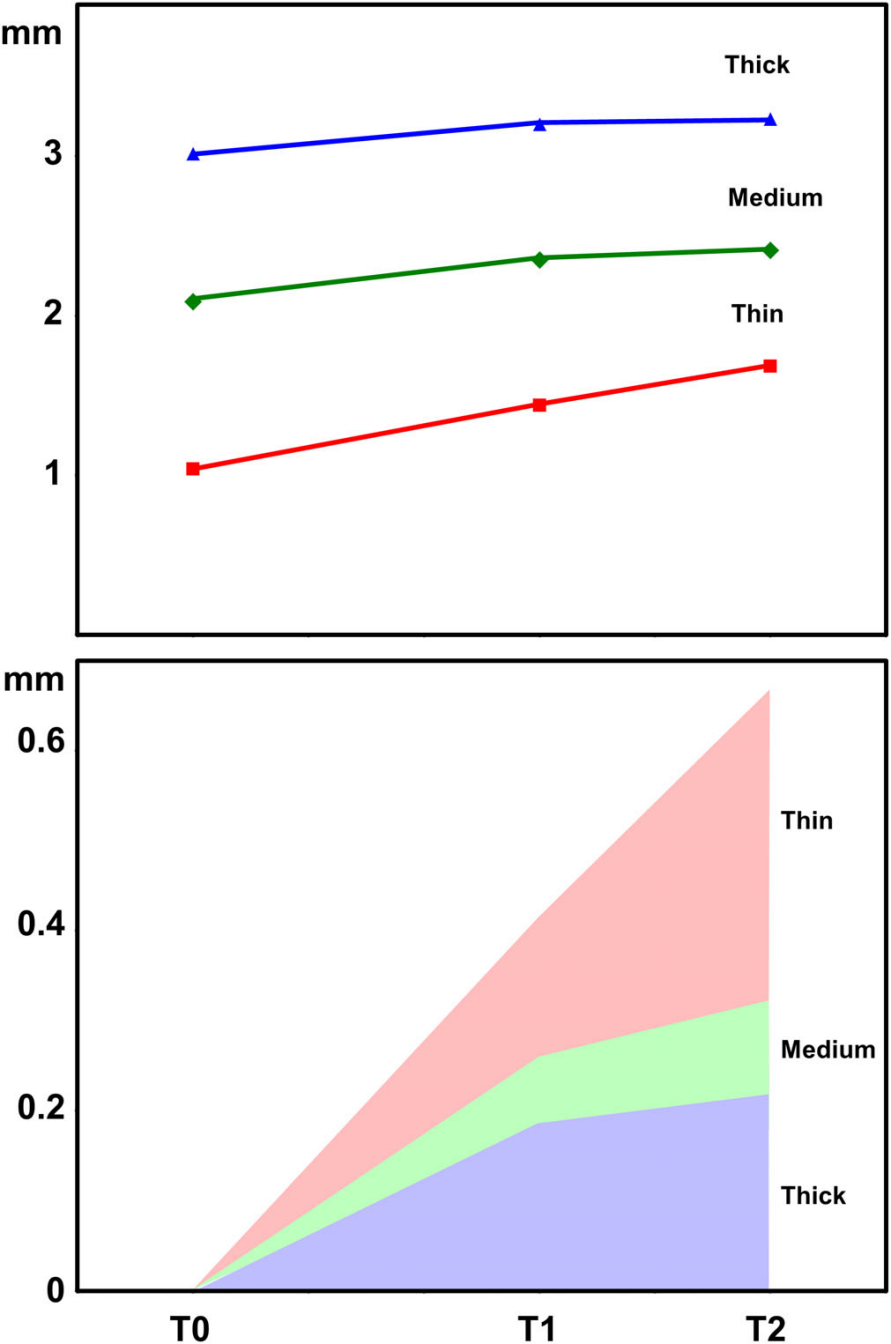
Above. Intra‐group comparisons showing PBLs significantly decreasing from T0 (baseline) to T1 (3 months) in all three groups. From T1 to T2 (5 months), PBL tended to stabilize in groups 2 and 3 (medium and thick mucosa), whilst a further notable decrease is present in group 1 (thin mucosa). Below. ΔPBL variations over time in all three groups. PBL, peri‐implant bone level

Intra‐group comparisons showed that PBL significantly decreased from T0 to T1 in all three groups. From T1 to T2, PBL tended to stabilize in groups 2 and 3 (medium and thick mucosa), whilst a further decrease in PBLs was recorded in group 1 (thin mucosa) (Table 1 and Figure 5).

**TABLE 1:** Peri‐implant bone level of implants of the three groups

	T0	T1	T2
Thin	1.038 ± 0.144	1.436 ± 0.282	1.682 ± 0.450
Medium	2.088 ± 0.135	2.349 ± 0.351	2.408 ± 0.389
Thick	3.014 ± 0.197	3.200 ± 0.339	3.232 ± 0.381

*Note*: Data expressed in mm (mean ± SD); T0, baseline; T1, after 3 months; T2, after 5 months.

PBL values recorded at each time point were converted into ΔPBL as follows:

T1 ΔPBL = T1 PBL – T0 PBL;

T2 ΔPBL = T2 PBL – T0 PBL.

ΔPBL variations over time are reported in Figure 5 and Table 2. Mean T1 ΔPBL was 0.41 ± 0.30 mm, 0.26 ± 0.40 mm, and 0.19 ± 0.35 mm in group 1 (thin mucosa), group 2 (medium mucosa), and group 3 (thick mucosa), respectively. No significant differences for mean T1 ΔPBL were demonstrated between the three groups after ANOVA test (*p* = 0.184). Mean T2 ΔPBL was 0.66 ± 0.49 mm, 0.32 ± 0.41 mm, and 0.22 ± 0.52 mm in groups 1, 2, and 3, respectively. Intra‐group comparisons showed no significant differences after ANOVA test between mean T1 ΔPBL and mean T2 ΔPBL in groups 2 and 3, whilst in group 1 the difference was only marginally significant (*p* = 0.075). Inter‐group analysis highlighted significant differences for mean T2 ΔPBL between the three groups after ANOVA test (*p* = 0.025).

**TABLE 2:** Peri‐implant marginal bone resorption at various time points

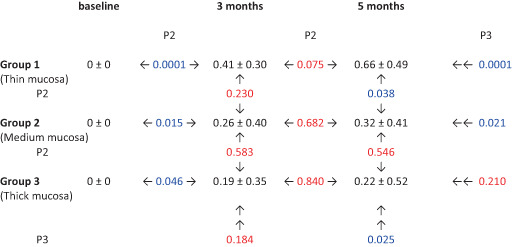

*Notes*: Data expressed in mm (mean ± standard deviation). *p*2 = probability after one‐way ANOVA test (2 groups). *p*3 = probability after one‐way ANOVA test (3 groups). Blue color indicates significant value (*p* < 0.05); red color indicates non‐significant value (*p* > 0.05).

The ΔPBL trend over time was also analyzed using simple linear regression and analysis of the coincidence of the regression lines (Table 3). The regression lines showed a significant direct relationship between ΔPBL and time (*p* < 0.05) for groups 1 and 2, but only a marginally significant correlation (*p* = 0.0871) for group 3. No significant differences were demonstrated comparing the intercepts of the three groups, whilst the slope inclination halved from group 1 to group 2 (*p* = 0.04), and became one third in group 3 with no significant difference between groups 2 and 3 (*p* = 0.55).

**TABLE 3:** Linear regression and coincidence analysis of PBLs

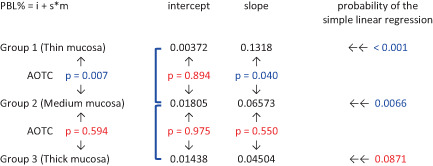

*Note*: Blue color indicates significant value (*p* < 0.05); red color indicates non‐significant value (*p* > 0.05).

Abbreviations: AOTC, analysis of the coincidence of the regression lines; i, intercept; m, months; PBL, peri‐implant bone levels; s, slope.

Overall analysis of the coincidence between groups 1 and 2 linear regressions (Table 3) showed significant difference in lines and slope inclinations (p = 0.007), whereas the comparison between groups 2 and 3 regression lines resulted in similar slope inclinations (*p* = 0.594).

When PBL variations are split into bone loss and bone remodeling, the present results show that at T2: around group 1 implants (thin mucosa) no BL was recorded and all 18 fixtures exhibited only BR; around group 2 implants (medium mucosa) one fixture out of 16 exhibited BL and 15 out of 16 exhibited BR; around group 3 implants (thick mucosa) 8 fixtures out of 15 exhibited BL and 7 out of 15 exhibited BR.

## 4 DISCUSSION

The present prospective study investigates if adaptation of apico‐coronal implant positioning in relation to mucosal thickness influences EMBL around tissue‐level implants during the first 5 months of healing. This evaluation was performed prior to crown delivery in order to eliminate multiple prosthesis‐related confounding factors. Multiple abutment connections/disconnections,^20^ abutment insertion timing,^43^ prosthetic abutment height,^21^, ^22^, ^23^, ^24^, ^25^, ^26^ abutment shape,^7^ crown emergence profile,^44^, ^45^ and adaptive response to occlusal loading^7^, ^46^ may all significantly influence further peri‐implant bone loss. The present study was designed to control additional anatomical and surgical variables with potential to influence marginal bone response. Specifically, a minimum of 6 mm of crestal bone width and 3 mm of keratinized tissue width were required at the implant insertion site; implant osteotomy was performed under abundant irrigation of cold saline solution in order to avoid bone overheating; a maximum peak insertion torque of 60 Ncm was established to prevent excessive interfacial pressure at cortical level potentially eliciting bone resorption^47^ and tissue‐level implants were selected in order to exclude all negative effects of an implant‐abutment micro‐gap positioned at or under bone level.

In presence of thick peri‐implant mucosa (>3.5 mm height), very limited marginal bone resorption (mean 0.19 mm) was recorded at 3 months, with a tendency to stabilize at 5 months (mean 0.22 mm). This finding suggests that, in presence of thick peri‐implant mucosa (>3.5 mm), very slight peri‐implant bone resorption occurs during supracrestal tissue height establishment, in accordance with numerous previous clinical studies.^31^, ^32^, ^48^

In presence of medium peri‐implant mucosa (between 2.5 and 3.5 mm), mean marginal bone resorption was slightly greater than in presence of thick mucosa, but followed the same trend over time (0.26 mm at T1 and 0.32 mm at T2).

Finally, in presence of thin peri‐implant mucosa (<2.5 mm), significantly greater marginal bone resorption was recorded at both time‐points (0.41 mm at T1 and 0.66 mm at T2). The vertical dimension of thin peri‐implant mucosa (<2.5 mm) was probably insufficient for adequate supracrestal tissue height establishment and wound healing consistently included marginal bone resorption, in perfect accordance with previous animal^29^, ^30^ and clinical studies.^31^, ^32^, ^48^, ^49^

However, two of the latter clinical studies^31^, ^48^ did not distinguish between the influence of surgical‐related factors, supracrestal tissue height establishment or prosthesis‐related factors on EMBL, as crestal bone changes were evaluated at implant placement and after a 1‐year follow‐up. The two other studies^32^, ^49^ performed a more detailed evaluation, taking radiographs at implant placement, prior to the prosthetic phase and after functional loading. Both studies concluded that, during supracrestal tissue height establishment, implants inserted in sites with thin mucosa showed significantly more bone resorption compared with implants placed in sites with thick mucosa. These outcomes are confirmed by the findings of the present study, in which mean bone resorption around tissue‐level implants from T0 to T2 resulted significantly different in the three (thin, medium, and thick mucosa) groups (*p* = 0.025).

No significant differences in PBL are present from T1 to T2 in all three groups, suggesting that marginal bone modifications related to supracrestal tissue height establishment occur mainly within 3 months after implant exposure to the oral environment. This finding is supported by a histologic human study by Tomasi and colleagues,^50^ showing that the soft tissue barrier around titanium implants was completely formed and developed within 8 weeks.

In the present study, apico‐coronal implant positioning was adapted in relation to mucosal thickness, in order to anticipate supracrestal tissue height establishment and avoid early exposure of the treated implant surface to the oral environment. This surgical strategy was successful as no BL was recorded at T2 in group 1 implants (thin mucosa), despite the fact that they presented the highest mean PBL reduction (0.66 mm). Only one group 2 implant (medium mucosa) exhibited BL at T2 (6.2%), whilst 53.3% of group 3 implants (thick mucosa) exhibited marginal bone resorption exposing the treated implant surface at T2. Although group 3 implants demonstrated the best results in terms of PBL preservation, their mean reduction in marginal bone level, albeit limited (0.22 mm), coincides with BL due to their equicrestal positioning. This finding suggests that also group 3 implants could benefit from a slight subcrestal positioning to prevent unwanted exposure of treated implant surface. The present results are in accordance with a recent meta‐analysis^51^ and with a previous study by Vervaeke and colleagues^52^ conducted on two‐piece implants with conical connection.

However, subcrestal implant placement may not be performed with all implant‐abutment connections. Numerous studies highlighted that flat‐to‐flat connections placed under bone level induce an inflammatory cell infiltrate as a defensive reaction to the presence of bacteria in the microgap between implant and abutment, resulting in increased peri‐implant bone resorption.^13^, ^14^, ^36^, ^53^ Bone resorption can be reduced by distancing the implant‐abutment junction from the bone, suggesting the existence of a spatial relationship between the inflammatory reaction occurring around the microgap and peri‐implant bone loss.^54^, ^55^, ^56^ In the present investigation, the use of tissue‐level implants eradicated this problem by moving the implant–abutment interface coronally, thus eliminating the detrimental effect of the microgap on peri‐implant bone stability.^37^, ^38^

This study presents some limitations that should be carefully evaluated when interpreting its clinical outcomes. Variations in apico‐coronal implant positioning could influence marginal bone remodeling irrespective of mucosal thickness: even if tissue‐level implants do not present a micro‐gap at or under bone level, further studies should clarify this point. This short term 5‐month evaluation does not consider the influence on EMBL of the subsequent prosthetic phases, which may significantly condition PBL. Specifically, abutment height may greatly influence further MBL after 6, 12, and 18 months of loading, irrespective of mucosal thickness, as demonstrated in numerous studies.^23^, ^25^, ^26^, ^57^, ^58^

The method used to measure mucosal thickness is potentially questionable due to the deformability of soft tissue. However, similar methods have been used previously in numerous studies^25^, ^26^, ^31^, ^32^ and currently, no scientific evidence confirms the superiority of other measurement techniques performed using ultrasonic devices.^52^ Moreover, data analyzed in the present study come from patients with specific characteristics, as stated in inclusion/exclusion criteria, and from a specific area (posterior mandible), limiting the generalizability of these results. Finally, PBL variations were measured on bi‐dimensional radiographs, which give no indication about buccal and lingual modification of peri‐implant bone.

Within the limitations of this study, it can be concluded that, during supracrestal tissue height formation, tissue‐level implants inserted adapting apico‐coronal positioning in relation to mucosal thickness exhibited greater marginal bone resorption at sites with thin mucosa than at sites with medium or thick mucosa. However, anticipating supracrestal tissue height establishment by adapting apico‐coronal implant positioning in relation to mucosal thickness may effectively prevent unwanted exposure of treated implant surface.

## AUTHOR CONTRIBUTIONS

**Sergio Spinato:** Concept/design; data collection; data analysis/interpretation; drafting article; approval of article. **Fabio Bernardello:** Data collection; critical revision of article; approval of article. **Teresa Lombardi:** Data collection; critical revision of article; approval of article. **Carlo Maria Soardi:** Data collection; critical revision of article; approval of article. **Marcello Messina:** Data collection; critical revision of article; approval of article. **Davide Zaffe:** Statistics; critical revision of article; approval of article. **Claudio Stacchi:** Concept/design; data collection; data analysis/interpretation; drafting article; approval of article.

## CONFLICTS OF INTEREST

The authors declare no conflict of interest.

## Supporting information


**Appendix S1** Supporting Information

## Data Availability

The data that support the findings of this study are available from the corresponding author upon reasonable request.
